# Supplementary material for: Behavioral contagion on social media: Effects of social norms, design interventions, and critical media literacy on self-disclosure
Source: PLoS One. 2021 Jul 19;16(7):e0254670. doi: 10.1371/journal.pone.0254670 (PMC8289004; doi:10.1371/journal.pone.0254670)
Supplement: Attachment — Submitted filename: Response to Reviewers.pdf [file pone.0254670.s001.pdf]

Response Letter

Dear Dr. Smith,
Dear Reviewers,

We would like to thank you for your thorough and thoughtful comments. We carefully addressed all of them. Please see our detailed responses to each of the reviewers' comments below. We highlighted the parts with more profound changes in yellow in the manuscript.

Please also note that the Open Science Framework (OSF) page related to this manuscript (https://osf.io/qxjsp/) was updated slightly as well (note that the current manuscript refers to study 2 (https://osf.io/yqhjr/) and 3 (https://osf.io/sxp2f/) within the broader project and the reproducible version of the manuscript as well as the respective online supplement is located in the folder "Manuscript 2 & Online Supplement 2" (https://osf.io/32zxe/). These pages contain a lot of additional information, including all data, scripts, and materials to reproduce/replicate the results/study as well as additional analyses. For some answers below, we refer to the relevant documents respectively.

Once again, thank you for your thoughtful comments. It is our hope that we addressed all of your comments satisfactorily.

Sincerely,
The Authors

Comments to by Reviewer #1 (R1)

R1.1: The communication and influence of social norms on computer-mediated environment is an interesting topic for scholars but is also a gap in the current literature. This manuscript used data from two experiments to address this gap with a focus on how perceived norms of disclosure on SNS affects self-disclosure behavior. The experiments were rigorously conducted. The presentation of theory and data was lucid. Overall, it is a solid piece of work that has potential to advance the online social influence literature. I have some minor observations as below, which I hope would help to improve the manuscript:

Answer 1.1 (A1.1): *Thank you very much for your overall positive evaluations and for your helpful comments and remarks. Below, we address each of them in more detail.*

R1.2: Page 2: "....In such a state of uncertainty, it is natural to look to other people's behaviors for guidance about appropriate disclosure." Is this conditioned by whether or not communicators' identities are anonymous? Perhaps, anonymity might reduce the influence of uncertainty on the link between norms - behavior. People might not be too concerned about the opinions and attitudes of others in the anonymity condition because their own identities are hidden and therefore, their need for social proof is low while social sanction is also unlikely. Several studies related to online incivility might provide some evidence to this possible effect. So, perhaps the authors should consider clarifying this argument a bit?

A1.2: *That's a very good point, and although the literature is more equivocal about the effects of anonymity and under which conditions anonymity creates more normative influence (see social identity and deindividuation effects (SIDE) theory by Spears, Postmes, Lea, & Watt, 2001), we agree with the reviewer about normative influence being higher when there is more risk of social sanctioning.*
Therefore, we have qualified our statement on page 2 and added a cite from Anderson & Dunning (2014) who discuss a similar point to say: "In such a state of uncertainty, it is natural to look to other people's behaviors for guidance about appropriate disclosure, especially in situations where the risk of social sanctioning is high, as in publicly observable behaviors" (Anderson & Dunning, 2014)."

R1.3: Page 2: Individual-norm types: Are subjective norms similar to injunctive norms? In the reasoned action approach, they seem to be used interchangeably. As the data showed these norms did not discriminate, if the authors operationalize norms as two dimensions of descriptive norms and injunctive norms, what would the CFA

results be like? In Study 2, there was a correlation r = .58, which was moderately strong and thus, norm types might still be discriminate?

A1.3: *The literature is indeed inconsistent with regard to how norm types are conceptualized or which labels are used. We decided to closely follow the comprehensive differentiation proposed in the literature review by Chung and Rimal (2016), who first differentiate collective (the actual prevalence of a behavior) and perceived norms, which they further distinguish into descriptive (perceived prevalence of the behavior), injunctive (perception of what people approve of), and subjective norms (perceived social pressure to conform). Empirical studies have shown that these norm types can differ in how they affect behavior (e.g., Park & Smith, 2007). Chung and Rimal though note that all norms may converge at times or at least can be highly correlated.*

With a lack of empirical evidence for how these norm types affect self-disclosure on SNSs, we preregistered individual hypotheses for each norm type in study 1 (H1a-H1c: https://osf.io/ufmnv/) and operationalized all three norm types based on the measure of Park and Smith (2007) (for item formulations, see: https://osf.io/kc5va/). We also preregistered to test the factorial validity of these scales. Results from these analyses can be found in the OSM (https://osf.io/rqft5/, p. 13 for fit indices, p. 15 for measurement models). The simple multidimensional model (Figure A) fitted well, but revealed very high inter-factor correlations (> .90). Treating them separately in subsequent models would have led to multicollinearity issues. We hence decided to go with a second-order model, which subsumed each norm type under a global factor, which we called the social norm. We believe that alternative models (e.g., a two dimensional model with the descriptive norm as one factor and the other two as another factor) are not feasible due to the high correlations between all three factors.

In study 2, we indeed could have followed our findings from study 1 and only preregistered one hypothesis (e.g., social norms positively affect visual disclosure on ESL) and assumed that the norm types would again converge into one global factor. Yet, we were unsure whether findings from study 1 would indeed apply to study 2 in which participants did not just see a screenshot of the news feed, but experienced a simulation in situ for 2 days. We reasoned that it is at least possible that they perceive some of the norm types differently. We hence preregistered again three hypotheses (study 2: https://osf.io/xb4pv/) and retested the factorial validity. Again, results from these analyses are reported in the OSM (https://osf.io/rqft5/, p. 28 for fit indices, p. 31 for measurement models). Indeed, this time, the inter-factor correlations were not as high, but still substantial. The second-order factor likewise fitted the data well with the subjective norm still having a substantial loading on the second order factor .68. To ensure consistency between the two studies, we decided to follow the measurement procedure of study 1 and used the global factor in subsequent analyses.

R1.4: Page 2-3: Definition of collective norms: Does the concept necessarily connect to a reference group? Rimal and Lapinski (2015) posit that collective norms operate at the societal level and that social identity works as a moderating factor in the norm-behavior link. The reference group likely involves social identification. I wonder if the reference group as operationalized in this study truly captures the population-level collective norms of users using the social media site as tested, unless the reference group is manipulated to saliently represent the large collective users. To be more specific, would the exposure to 50 posts as presented in this study capture the actual code of conduct of a large population of users? If not, the causal effect of collective norms on normative perception as currently claimed might not be clear. Perhaps, mentioning that participants observing other users' posts and comments would suffice as you did on page 4, line 163.

A1.4: *We modelled our conceptualization and operationalization of collective norms on Rimal and Lapinski's (2015) arguments that "collective norms operate at the societal level or at the level of the social network" (p. 395), contrasting them with perceived norms that operate at the individual level. Cautioning against the operationalization of collective norms as a mere aggregation of individual-level perceived norms, Rimal and Lapinski espouse that "an aggregation of individuals' behaviors, however, can serve as a proxy for collective norms. The sum total of individual behaviors in a community is a good indicator of the collective norm in that community" (p. 396). Furthermore, they used the proportion of people in a bounded community, as defined by researchers, to operationalize collective norms, which was precisely our approach to manipulating collective norms by using the proportion of visual disclosures in a newsfeed, i.e., a bounded community participating in that specific social network site. We have clarified in the manuscript that our approach is consistent with Rimal and Lapinski's (2015) approach to collective norms in a bounded community and checked the consistency of terminology throughout the manuscript.*

R1.5: Page 3, line 109: "there may be other factors..."

A1.5: *Thank you, we corrected the sentence accordingly.*

R1.6: Page 6, line 213, remove bracket.

A1.6: *Removed accordingly.*

R1.7: What were the differences between posts and comments? How were the comments created and what did they say? Were there possible effects of these comments in the experiment?

A1.7: *EatSnap.Love (https://eatsnap.love/login) in combination with Truman (https://github.com/difrad/social_norms_truman) allows researchers to simulate an entire social media use experience. When study participants thus sign up on the page and enter, they can build a profile and browse a social media feed with posts. From the very beginning, we decided to include comments and likes underneath each post to make it more realistic and similar to the existing social network sites. Throughout the simulation, new posts and subsequently new comments and likes appeared in the feed. It is important to note that each participant had exactly the same experience, with regard to sequencing of posts in a newsfeed based on the time they entered the site. For example, if a participant enters EatSnap.Love for the first time at 12.00am, a new post for example could happen 3 minutes later. Some comments might appear seemingly randomly after several minutes. Another participant who enters the site for the first time at 4.00pm, likewise sees the exact same new post 3 minutes later and the exact same comments appear again after several minutes.*

The entire simulation (including users with their individual biographies, names, and profile pictures; posts by these users including images and captions, as well as comments by other users and like patterns) was carefully designed in iterative steps. Several research assistants, as well as the first and second authors, crafted the simulations. After several pretests, the simulation was deemed sufficiently realistic (as evidenced also by the perceived realism analyses reported in the paper). Two example posts are shown in Figure 1 in the paper. All posts and their meta data (including e.g., captions and when they appear) can be assessed here: https://github.com/difrad/social_norms_truman/blob/master/input/new_posts.json. All comments and their meta data can be assessed here: https://github.com/difrad/social_norms_truman/blob/master/input/new_comments.json. An example comment is e.g., "dude this photo is awesome. you should get whoever took it to join here haha". Overall, posts and comments were relatively positive.

The two studies used similar posts and comments, but study 1 exposed participants to only screenshots of the news feeds without immersing them into an actual SNS-like experience. Furthermore, the news feeds in Study 2 were longer in length, with more posts and comments, to account for a two-day study duration and create an authentic SNS-like experience. Across the experimental treatments for both study 1 and study 2, the content and the number of captions and comments were identical, except for the differences in visual disclosures, as prescribed by the experimental manipulation.

R1.8: Measures: Can you add a few sample items for other norm types?

A1.8: *Please note that all items for study 1 (https://osf.io/kc5va/) and study 2 (https://osf.io/5fph7/) are available via the respective Open Science Framework (OSF) page. All item formulations and respective descriptive analyses are further also included in the Online Supplement (https://osf.io/rqft5/, for study 1, see p. 6; for study 2, see p. 27). That being said, we now included one example item for each norm type in the manuscript.*

R1.9: Can you please explain why the 2 items measuring critical thinking were removed? Is it because of low factor loading (e.g., below .40)?

A1.9: *It is important to note that we created a new measure for critical thinking deposition based on several existing scales (the cognitive reflection test, the critical thinking disposition scale, and the need for cognition scale). The two items that we removed stem from Cacioppo et al.'s need for cognition scale. In the confirmatory factor analyses and subsequent investigations of the modification indices, these items did not have low factor loadings, but appeared to form a singular factor. By removing the two items, the Chi-Square value could be*

reduced by a value of 201. As we considered the remaining items still a good representation of the concept of interest, we decided to remove those two items in favor of a better fitting uni-dimensional model. All of these analyses are reported and accessible in the analysis output shared on the OSF (https://osf.io/37en4/; please note that you need to download the html file to be able to view it properly). Due to the manuscript space restrictions, we decided to not report these scale adjustments in more detail in the manuscript. If the editor and reviewer think they should be discussed in the paper, we are of course happy to do so.

R1.10: In Study 1, it looks like you emphasized the literature method gap of not measuring actual behavior, but Study 1 did not account for this gap. You only did so in Study 2. So, would it be more appropriate to move the argument to the front end and explain in Study 1 that you aimed at examining norms-likelihood of self-disclosure (or behavioral intention as you wrote on page 8, line 328) as the first step of the project?

A1.10: *Thank you for this comment. With this project, we attempted to close several gaps in the literature. One goal was to experimentally study norm effects and thereby manipulate the collective norm. This goal was addressed in study 1 and 2, hence its position in the overall manuscript. The second goal was to observe behavior (i.e., visual self-disclosure) in a dynamic and realistic setting, rather than use self-reports. We do understand your suggestion to move the second goal entirely to the description of study 2, but we would like to emphasize that the theoretical rationale and literature presented in the beginning pertains to both studies, with study 2 building consecutively on study 1. Please note that we clearly state the difference already in the introduction (p. 2, lines 32-42). To make this even clearer, we added the following sentence in the method section on p. 5, lines 183-187:*

> *"In a first step, Study 1 presented participants with a snapshot of the simulation, which contained about 50 posts and respective comments, and investigated people's subsequent disclosure intentions. In a second step, Study 2 (see further below) implemented the fully operational simulation over a 2-day period and investigated people's actual behavioral adaptation."*

… and we further emphasized the different goals per study by slightly changing the first sentence when introducing study 2:

> *"With Study 2, we wanted to test whether the results from Study 1 could be replicated in a simulated social media environment, in which we could observe actual behavioral adaptations to the norm prominence in situ.*

R1.11: Other than that, I feel that the manuscript is clear and a pleasure to read. The recommendation for future research is very informative. I'd also recommend emphasizing the specific social interactive network platforms because research has shown that heuristic features such as like, share, and comments might work differently depending on different platforms and user groups. Thank you for the opportunity to read this qualified work

A1.11: *We once again thank the reviewer for their positive evaluations and valuable comments for improvement. We hope we addressed all of your comments satisfactorily.*

Additional references

Chung, A., & Rimal, R. N. (2016). Social norms: A review.Review ofCommunication Research,4, 1–28.https://doi.org/10.12840/issn.2255-4165.2016.04.01.008

Park, H. S., & Smith, S. W. (2007). Distinctiveness and Influence of SubjectiveNorms, Personal Descriptive and Injunctive Norms, and Societal Descriptive andInjunctive Norms on Behavioral Intent: A Case of Two Behaviors Critical toOrgan Donation.Human Communication Research,33(2), 194–218.https://doi.org/10.1111/j.1468-2958.2007.00296.x

Rimal, R. N., & Lapinski, M. K. (2015). A re-explication of social norms, ten years later. Communication Theory, 25(4), 393–409. https://doi-org.vu-nl.idm.oclc.org/10.1111/comt.12080

Spears, R., Postmes, T., Lea, M., & Watt, S. E. (2001). A SIDE view of social influence. In J. P. Forgas & K. D. Williams (Eds.), Social influence: Direct and indirect processes (pp. 331–350). Psychology Press.

Comments by Reviewer #2:

Reviewer(R)2.1: The authors present a duo of consecutive and complementary studies investigating the effects of social norms on intended and actual disclosure behaviors on a fictional social media site. The first study tests intentions to visually disclose on the site based on reviewing approximately 50 posts. They find a relatively small percentage (20%) of posts are enough to influence perceptions of social norms encouraging visual disclosures. Additionally, the critical media literacy subconstruct of Deliberation moderates the effect of social norms on intentions to visually disclose. The second study builds on this by inviting participants to spend two days using the platform and captures their activity – primarily visual disclosures. While the findings with social norms is replicated, Deliberation was not a significant factor in Study 2.

Most of my research focuses on online self-disclosure, so I am quite familiar with this area of study. I've also worked with MTurk for data collection. I currently have more experience with survey methods than experiments, but I am familiar with experiments.

Overall, I enjoyed reviewing this research study. I found the research questions and the approach to the study interesting. The paper is quite polished in terms of writing, with only a handful of minor errors. The discussion is reasonable and doesn't overextend the findings of the two studies. I do think there is room for improvement, though many of my recommendations are to help make parts of the manuscript clearer and to add some additional detail in other places. The points below are organized by my perception of most pressing to address.

Answer(A)2.1: *We thank the reviewer for their positive evaluation and valuable comments. Below, we addressed all of your comments in detail.*

R2.2: Why was Study 2 only over two days? What evidence indicates this would be sufficient time to elicit an adequate number of disclosures? It seems very odd for it to be so short, and thus unsurprising that most participants only had a single visual disclosure.

A2.2: *The use of a simulated environment allowed us to go beyond a single-shot exposure experiment. To increase realism and the length of exposure, we decided to expand it over 2 days, rather than a single day. However, we had to balance realism with practical considerations (participants' compensation, the number of posts (~550 posts across all the experimental conditions) we had to create in order to have an adequate newsfeed simulation with over 145 posts for each day, etc), which is why we decided to go with only 2 days. We acknowledge low variance as a limitation in the paper, however. At the same time, in terms of eliciting the adequate number of disclosures, a single post with participants' visual disclosure per this period of time is consistent with the frequency of posts in real social media sites. For example, French and Bazarova (2017) found that participants posted an average of 2.45 posts (SD=2.02) per person over the course of 5 days looking across three different social network sites: Facebook, Twitter, and Instagram.*

R2.3: Why was ANOVA the chosen statistical method for Study 1? Given the nature of the data, SEM or regression (as in Study 2) seems more appropriate. It isn't clear why ANOVA was selected or what benefit it provides over other statistical approaches.

A2.3: *We very much empathize with the reviewer's comments as, from our point of view, analysis of variance (ANOVA) and respective regression-based approaches indeed are conceptually similar and do not offer particular advantages over each other. In the media psychological literature, however, ANOVA is often the most chosen approach to analyze experimental data. To align with this tradition, we chose to analyze the data for study 1 accordingly. As inferences drawn from either method should not differ, we are hesitant to change it, particularly because we preregistered to use ANOVAs rather than regression models. For study 2, we chose a logistic regression model as it is likewise the most commonly used approach to analyze data when the outcome variable is binary (in our case 0 = no disclosures, 1 = visual disclosure). We again preregistered this approach and are likewise hesitant to change it at this point.*

The reviewer is right to suggest structural equation modelling (SEM) as an alternative, again regression-based approach. Although SEM could improve the estimation slightly due to the accounting for measurement error, we believe that the added benefit is comparatively small given the highly reliable measures (see CFAs and measurement models in the OSM). A second reason for not using SEM is related to the moderation analyses. As some of our hypotheses (in both studies) describe potential interaction effects, we decided to not use latent modelling as interaction analyses with latent variables are not trivial and often run into convergence or estimation problems. In sum,as we do not expect different inferences by using different methods, our preference would be to stay with the preregistered analyses.

R2.4: Study 1 uses nine different groups and Study 2 uses four groups based on the combination of manipulations. The authors don't address in the manuscript if these different groups are statistically similar to each other. As such, it isn't clear if there are other factors that can be attributed for the differences observed.

A2.4: *Thank you for this comment. We actually did test whether groups are statistically indifferent from each other (both in study 1 and study 2). All these analyses are reported and accessible via the explicitly linked Open Science Framework (OSF) project page. For example, you can find all analyses for study 1 here: https://osf.io/37en4/ (please note that you have to download the html file to view it properly). Under 3.5, you will find descriptive analyses per condition as well as relevant difference tests. A chi-square test suggested that sample sizes between the nine conditions did not differ significantly ($\chi^2(4) = 1.15$, $p = .887$. Age, gender, and education likewise did not differ across the conditions (see relevant output under 3.5). Likewise, for study 2 (https://osf.io/xhefu/, section 4.3), a chi-square test suggested that sample sizes between the nine conditions did not differ significantly ($\chi^2(1) = 0.73$, $p = .393$). Age, gender, and education likewise did not differ across the conditions (see relevant output under 4.3). We hence assumed that the randomization was successful in both studies. Due to space restrictions, we decided to report these analyses only in the shared output and not in the manuscript. We nonetheless added the following sentence to both study 1 and 2:*

> *"Groups did not differ in sample size, age, gender, or education. The randomization was hence deemed successful."*

We could, of course, add all test results to the manuscript, but we believe that this would make the method section less readable and the overall manuscript longer.

R2.5: In both studies while discussing the collective norm effect, I find myself confused. After some reflection, I've determined it's because I was thinking of the collective norm and the social norm perceptions as being the same constructs. I don't readily associate the manipulations in the study with the term collective norm. Even in studying Table 1, I wasn't associating "Number of Identifiable Posts" with collective norm or the manipulation. I know the authors spend some time identifying the different social norms on Page 2, but collective norms receive comparatively little discussion. It gets challenging to keep up with the different norms, especially when they're all social norms and that term is most often only used in reference to perceived norms. I think consistency would help prevent this confusion, among other clarifying strategies.

A2.5: *Thanks for this point. We have addressed this point in response to Reviewer 1's question (please see A1.4). Specifically, we clarified the difference between collective norms and perceived norms based on Rimal and Lapinski's (2015) conceptualization and operationalization of collective norms. Following your suggestions, we have also expanded the description of collective norms in the theory section, the description of the operationalization of the collective norm in the methods sections, the row labels in the table, as well as the discussion of collective norms to make more clear how they differentiate from the other norms examined in our study. Please note that the term collective norm is also inserted in brackets in all the respective hypotheses. Most importantly, we added the following sentence to the method section of the first study:*

> *"We manipulated the collective norm by varying the amount of visual disclosure in posts and the profile pictures."*

And emphasized the following sentence in the methods section of study 2:

> *"We manipulated both the prevailing collective norm related to visual disclosure in posts and the design of the SNS to include a privacy nudge."*

We hope that this clarifies the distinction between the collective norms (our manipulations) and the perceived norms (post-manipulations measurements).

R2.6: Why was the same perceived norm scale used in Study 2? Alternatively, why were the three perceived norms hypothesized separately in Study 2? Study 1 provided evidence that the selected measure lacked discriminant validity for the three subscales, so it seems illogical to do the same thing and expect different results. I believe this needs to be addressed directly either with the hypothesis or the measure discussion. That said, I appreciate the discussion at the end of the paper and proposed future research regarding this issue.

A2.6: *We do understand the reviewer's suggestion in this regard. Please note that reviewer 1 made a similar comment (R1.3). Our answer to his/her comment already explains some of our reasoning, which we would like to expand here. On the one hand, the literature (e.g., the comprehensive literature review by Chung & Rimal, 2016) suggest that the three different norm types are conceptually different, but may overlap or converge in some scenarios (or with regard to some behaviors) and not in others. Given the differences between the two studies – study 1 used screenshots and examined disclosure intentions, whereas study 2 used a 2-day simulation and examined actual behavior, we were indeed uncertain if results (even the measurement models) from study 1 would entirely replicate in study 2. Indeed, in our previous project, which also used the same platform (ESL and Truman), results differed greatly between the first screenshot-based study and the second study using the actual, fully-developed simulation (Taylor et al., 2019). In fact, the CFAs conducted to test the factorial validity in the present studies suggest some small differences between study 1 and study 2 (see links in our answer to R1.3), but did not lead to different decisions with regard to how to operationalize norms. Furthermore, we are hesitant to change our preregistered hypotheses after the results are known, but do agree that this could be made clearer and hence adapted the method section in which we describe the CFAs and our reasoning.*

R2.7: The manuscript presents three research questions on page 4 – well into setting up and discussing the first study. I generally prefer research questions to appear earlier in the paper (i.e. the introduction) because I view these as big picture questions that inform the model (and hypotheses by extension) and research design. It's confusing to me that these are presented after a hypothesis, particularly as the text leading up to the RQs seems to indicate a specific hypothesis – before the authors seem to lose confidence and indicate there's insufficient extant research to support a specific hypothesis. I think presenting the research questions earlier (especially as they relate to both studies) and then discussing these other avenues of exploration as ways to address the research questions would be more direct and allow the research questions to serve as overall guides for the manuscript; this also allows the authors to speculate about the possible relationship based on what is known without forming specific hypotheses.

A2.7: *We believe there is a misunderstanding with regard to how we view and understand specific RQs. They do not serve as overall guides to the manuscript and thus do not represent big picture questions. Such research guiding statements are made in the introduction, when we discuss the goals of the paper (p. 2, lines 30-43). The research questions, in contrast, refer to specific relationships or effects that we wanted to test in each of the studies, but a lack of empirical evidence made it hard to derive precise predictions. So instead of formulating hypotheses, we pose non-directional questions that are nonetheless testable and specific with regard to the relationship or effect of interest. This is also why their justification is somewhat similar to the justification for hypotheses. We do acknowledge that there may be disciplinary differences in how research-guiding questions, hypotheses and specific research questions are used and distinguished. We hope that our response provides more clarity in this regard.*

R2.8: It isn't clear if the likelihood of disclosing oneself scale is self-developed. If it is, why were existing scales not used/modified and what pretesting was done to validate the measure?

A2.8: *We agree that prior research has used and developed a variety of self-disclosure scales. In our own work, we have previously adopted such scales (e.g., the self-disclosure scale by Wheeless & Grotz, 1975). For this particular project, however, we identified the need for a more specific scale that a) specifically focused on visual disclosure and b) aligns closely with the way we measured norm perceptions (principle of compatibility, e.g., Ajzen, 1991). We believe that the scale has a high face validity, but please note that we tested the factorial validity and reliability of the scale comprehensively as well. The scale fitted the data exceptionally well and also had a high composite reliability (Chi-Square(5) = 15.89, p = .007; CFI = >.99; TLI = .99; RMSEA = .06, SRMR = .01; McDonald's omega = .97). Please note that all item formulations (p. 8) and results from the confirmatory*

factor analyses (p. 13 for fit indices, p. 15, for the factorial models and factor loadings) are presented in the OSM (https://osf.io/rqft5/). That said, we indicated more clearly in the manuscript that the scale was self-developed.

R2.9: Relatedly, it isn't clear if the 20-item pool of critical media literacy items was self-developed or drawn from the referenced studies. Additionally, it isn't clear if the deliberation subscale was added to the 20-item pool or counted within it. The language in this part just needs some clarifying.

A2.9: *We agree that the description was confusing. We indeed developed a new 20-item pool based on three critical media literacy scales AND the deliberation subscale. Respective factor analyses can be found in the OSM (https://osf.io/rqft5/, p. 10-14). We hence improved the section and believe that it is now more clearer. Please also note that all items can be found in the study's codebook (https://osf.io/kc5va/, p.11). Here, it is also indicated which items were adapted from which original scale.*

R2.10: Section 4.1, Lines 581-583, the authors discuss visual disclosure in Study 1 as though it represents behavior and is therefore directly comparable to the actual behaviors captured in Study 2. However, my understanding is that Study 1 asked respondents how likely they would be to post information on the simulated site after reviewing several posts; I interpret this as intention to act in a certain way, not actual behavior. As such, it may be that Deliberate Media Use is a significant predictor of intention but not behavior, which is why it's significant in Study 1 but not Study 2. I agree that low variance may also be a factor in detecting small effects, but I also think it's important to consider what exactly has been captured in each study regarding visual disclosure.

A2.10: *We appreciate this explanation and have revised this section appropriately. Specifically, we now acknowledge that differences in the measurement of self-disclosure (intentions vs. actual behavior) between study 1 and 2 may explain why the moderating effect of critical media literacy was not found in study 2. Thank you for comment.*

R2.11: Beginning in Study 2, the authors reference SNS. To this point – with the exception of the abstract – the authors have referred to social media. These are not interchangeable concepts (SNS is the more narrowly defined of the two). Considering Instagram and Twitter are the referenced sites for the simulated environment, it may be inaccurate to call it an SNS – Twitter is a microblog, not an SNS, and it seems like the experimental setting may mimic this more because friend lists (a key component of SNS) are not mentioned or seem likely to be supported.

A2.11: *We agree that the broad framing was inappropriate. We have revised the entire manuscript in this regard and focus on social network sites (SNS). While we agree that friends lists are a defining feature of SNSs, the look and feel of EatSnap.Love, despite not featuring a friends list, is still more like an SNS (in particular, Instagram). The reference to Twitter was definitely misleading here, which is why we deleted it.*

R2.12: To that last point, it isn't clear why relationship management was included as a perceived benefit. Relationship management indicates that there's a pre-existing relationship to nurture. This simulated environment isolates users so they only interact with bots so – by extension – they don't know the other "users" and therefore can't have pre-existing relationships to manage. It seems illogical to include.

A2.12: *Although we understand the reviewers' point, we politely disagree with his/her conclusion. First, many studies in the area of privacy research have shown that concerns about and perceived benefits of using SNS generally explain a considerable amount of variance in self-reported self-disclosure on such platforms (also referred to as the privacy calculus). In our study, we wanted to control for such factors to clearly parcel out the true effect of norms. These variables refer to general expectations towards using SNS, which users form based on previous SNS experiences and may transfer to novel SNS experiences such as our platform. Second, we used the perceived benefits scale (consisting of multiple dimensions, including enjoyment, relationship initiation/maintenance, and self-presentation) by Krasnova et al. (2010), who explicitly investigated this privacy calculus on SNSs. The subdimension focusing on relationships does not only focus on relationship maintenance, but also on people's perception of how well they can get in contact with new people using SNSs (see item formulations on p. 7 in the study codebook: https://osf.io/5fph7/). One item particularly reads as follows: "Social media is useful for developing relationships with people (business or private)". We therefore feel that it is*

reasonable to assume that people who generally believe that SNS are useful for relationship building and maintenance, will be more likely to disclose themselves in a novel environment as a potential venue for exploring and building online relationships.

R2.13: The authors state that posts containing children and other family members were coded as identifiable and then explains why this kind of photo deidentifies the target individual. First, this discussion makes me question if there's a typo – e.g. that the photo wasn't coded identified because more people in the picture deidentifies the target person – so I think that should be revisited for clarity. Second, it's noted that this differed from the pre-registration of the study but it isn't explained why.

A2.13: *Thank you very much for this comment. It was indeed a typo, which we have corrected. In our preregistration, we indicated the following:*

> *"Our primary dependent variable will be people's posting behavior on ESL during the two-day period. We therefore will code the photos that participants have posted (1 = participant is identifiable, 0 = participant is not identifiable).*

When coding the actual disclosures of participants, we realized that some participants posted pictures of their children or family members. We deemed these types of visual disclosures on par with visual disclosures containing the discloser as they likewise identify the user and hence decided to code these posts as "identifiable" as well. Hence the derivation from our preregistration. We made this more clearer in the manuscript.

R2.14: I think a table summarizing the hypotheses and results by study (i.e. hypotheses on the rows and studies on the columns) would be useful going into the final discussion.

A2.14: *Due to space restrictions, we believe adding another table is not possible. That said, we tried to make it clearer throughout the manuscript which hypotheses were supported and which not. If the editor and reviewer still think an additional table is necessary and additional space is provided, we are of course happy to include it.*

R2.15: In Study 1, participants were excluded for spending less than 10 seconds on the simulated feed; how did the authors determine how long participants spent on the simulated feed?

A2.15: *We used qualtrics for this experiment. As the feed was embedded in a separate page in the survey, we could easily assess the duration participants spent on this site, as qualtrics provides the duration in seconds for each page of the questionnaire. The data on OSF contains a respective variable.*

R2.16: Similarly, in Study 2, it's stated that participants were asked to spend at least 5 minutes on the platform each day. How was this tracked? What was the average time spent on the site each day?

A2.16: *This was actually not tracked or controlled, but rather an instruction that we presented to the participants before the study. What we did track, however, was the amount of times people visited the site. On average, participants visited the site M = 7.88 times per day (SD = 6.71). We added this information to the description of study 2.*

R2.17: The authors do a fair job justifying use of an MTurk sample. However, there are several studies that raise valid concerns with collecting data on MTurk – including the problem of professional MTurkers. I didn't see any formal limitations of the study discussed, but it might be worth at least mentioning using a different sample in future research to avoid potential biases caused by only using MTurk samples.

A2.17: *We agree and added a respective limitation to the manuscript as well as referencing an overview chapter on common concerns and problems with MTurk samples.*

R2.18: Section 3.3.1, Lines 391-393, the authors mention using the "same cover story," but no cover story was

discussed for Study 1.

A2.18: *Thank you for pointing this out. We added a short description of the cover story to the study 1 method section as well. Please note that the entire cover stories can also be accessed on the OSF page (https://osf.io/kc5va/).*

R2.19: The authors make a statement about the shortcomings of cross-sectional surveys that is uncited (Section 2.4, Lines 159-161).

A2.19: *This statement is based on our own analysis, which is why we did not include any reference. We believe it to be generally true that cross-sectional surveys only allow for correlational analyses based on self-reported measures – in the case of norm-behavior links, they only allow to study relationships between self-reported norm perceptions and self-reported behaviors. Although this can of course be informative, such a design cannot investigate causality and more specifically, cannot measure the collective norm independently of individual perceptions. As mentioned earlier, Rimal and Lapinski (2015) caution against operationalizing collective norms as a mere aggregation of individual-level perceived norms. We hence believe that our study indeed moves beyond limitations of cross-sectional survey studies in this respect.*

R2.20: Figure 1A is quite blurry, so it's difficult to see what exactly is going on. It might be worthwhile making A and B separate figures (especially as 1B is only relevant to Study 2) so that the images of 1A can be slightly larger and hopefully clearer.

A2.20: *Thank you for pointing this out. We separated the figures and increased the resolution. We hope it is now clearer.*

R2.21: Very minor editing. The most consistent error is not spelling out numbers zero through nine. There are also a couple of times within the Study 2 section where a comparison is being made to Study 1 but the authors have said Study 2 instead (e.g. Lines 433 and 438).

A2.21: *Thank you for catching those issues. We corrected them accordingly.*

R2.22: Separately – although I don't think much can be done about it now – I wanted to raise my concern with participant compensation in this duo of studies. In both studies, the participants seem grossly underpaid for their participation. MTurk typically recommends paying participants the equivalent of minimum wage in the United States ($7.25 per hour). While the authors don't specify in the manuscript if a location filter was used, the compensation on the first study is $2.50 an hour for the average participant. While payment looks better for Study 2 ($10 total), the average time to complete the pre- and post-surveys was not discussed, so it's harder to judge if compensation was reasonable. There isn't much that can be done on this point now, but I wanted to raise this issue because I find it unethical to so poorly compensate participants.

A2.22: *This is a very important point. We were new to MTurk and followed conventions that we saw for other MTurk studies at that time. We were also fully transparent with our participants about the amount of compensation for their participation. Having said that, we acknowledge that this payment was below the acceptable threshold, and we have definitely learned our lesson and have been using the equivalent of minimum wage or higher in all of our subsequent MTurk studies.*

Additional references

Rimal, R. N., & Lapinski, M. K. (2015). A re-explication of social norms, ten years later. Communication Theory, 25(4), 393–409. https://doi-org.vu-nl.idm.oclc.org/10.1111/comt.12080

Taylor, S., DiFranzo, D., Choi, Y. H., Sannon, S., and Bazarova, N. (2019). Accountability and empathy by design: Encouraging bystander intervention to cyberbullying on social media. Proceedings of the ACM on Human Computer Interaction Journal (PACM CHI Journal), 3, 1-26
